# Longitudinal ^1^H NMR metabolomics of serum in a chronic temporal lobe epilepsy model reveals systemic metabolic remodeling

**DOI:** 10.3389/fmolb.2026.1882162

**Published:** 2026-08-31

**Authors:** Veronica Ghini, Lucrezia Cosottini, Miriana Scordino, Nicolò Ricciardi, Giulia Urone, Giuditta Gambino, Valentina Di Liberto, Paola Turano

**Affiliations:** 1 Department of Chemistry, University of Florence, Sesto Fiorentino, Italy; 2 Department of Biomedicine, Neuroscience and Advanced Diagnostics (BIND), University of Palermo, Palermo, Italy; 3 CERM, University of Florence, Sesto Fiorentino, Italy

**Keywords:** ^1^H NMR, animal models, epilepsy, lipoproteins, metabolomics, pilocarpine

## Abstract

**Introduction:**

Temporal lobe epilepsy is associated with systemic metabolic alterations that may evolve during disease progression and in response to pharmacological treatment. This study employed a longitudinal ^1^H NMR-based metabolomics approach to characterize the serum metabolic profile of a chronic pilocarpine-induced rat model of temporal lobe epilepsy.

**Methods:**

Serum samples were collected longitudinally to identify phase-specific metabolic signatures. Following phenobarbital administration, animals were classified as drug-sensitive or drug-resistant according to their treatment response. The resulting metabolic profiles were investigated using multivariate and univariate statistical analyses.

**Results:**

The analysis revealed progressive systemic metabolic remodeling characterized by extensive alterations in lipid-related signals and significant increases in the ketone bodies 3-hydroxybutyrate and acetoacetate, as well as in glutamine. Following phenobarbital treatment, OPLS-DA showed a strong metabolic similarity between the drug-sensitive and drug-resistant groups, which occupied nearly identical metabolic spaces. Nevertheless, after stratification, creatine emerged as the only significantly different metabolite between responders and non-responders (p = 0.001).

**Discussion:**

The convergence of the metabolic profiles of drug-sensitive and drug-resistant animals suggests that the systemic effects of chronic epilepsy and phenobarbital treatment dominate the serum metabolome, potentially masking subtle molecular signatures associated with drug resistance. Creatine represents a notable exception and warrants further investigation as a potential marker distinguishing responders from non-responders.

## 1 Introduction

Temporal lobe epilepsy (TLE) is a multi-scale systemic disorder linking metabolism, circuits, and excitability, characterized by profound biochemical remodeling (Rho and Boison, 2022). Seizure activity in the temporal lobe triggers widespread alterations in metabolism, neurotransmission, inflammation, and neural network organization, reciprocally reinforcing each other, highlighting the global disease impact (Chen et al., 2023; Auer et al., 2026). Interestingly, alterations in brain metabolism may not only arise as a consequence of epileptic seizures, but also actively contribute to exacerbating their detrimental effects, thereby establishing a self-reinforcing cycle associated with recurrent seizure activity (Rho and Boison, 2022).

Despite the availability of numerous anti-seizure medications, approximately one-third of patients remain drug-resistant, and the underlying molecular mechanisms of epileptogenesis, the process by which a healthy brain becomes epileptic, and epilepsy progression remain partially elusive (Xu et al., 2022). In this context, metabolomics emerges as a transformative tool, enabling the identification of pathway-level dysregulations and functional tissue biomarkers associated with neurological disorders. As the closest functional representation of the phenotype, the metabolome captures the dynamic interplay between genetic predisposition, epigenetic modifications, and environmental factors (Griffiths, 2008). Unlike genomics or proteomics, which indicate potential or catalytic capacity, metabolomics provides a “real-time” readout of the cellular physiological state (Griffiths, 2008). The adoption of an untargeted metabolomics approach is particularly crucial in epilepsy research. Rather than focusing on a predefined set of molecules, an untargeted strategy allows for the unbiased global profiling of thousands of small molecules (Vignoli et al., 2019). This “discovery-based” methodology is essential for identifying novel biomarkers, thus uncovering unexpected metabolic signatures that traditional hypothesis-driven research might overlook. The ability to map altered metabolomic profiles might help elucidate the complex cross-talks between neurotransmitter imbalances, mitochondrial dysfunction, and neuroinflammatory cascades.

The ultimate goal of applying metabolomics to animal models is to foster a “bench-to-bedside” transition. Animal models allow for controlled longitudinal studies and direct access to brain tissue, providing a high-resolution map of the disease’s molecular roots. However, for these findings to have clinical utility, they must be translated into minimally invasive diagnostic tools. The identification of circulating metabolic proxies in blood (plasma or serum) represents the bridge between experimental discovery and clinical application. By aligning the metabolic fingerprints found in preclinical models with those observed in patient cohorts, one can move toward more clinical interventions, where metabolic profiling guides therapeutic monitoring, and the development of targeted, disease-modifying treatments.

Here, we exploited a longitudinal within-subject design to characterize systemic metabolic changes across epileptogenesis and TLE progression in a pilocarpine-induced rat model. By leveraging the high reproducibility of ^1^H NMR spectroscopy, we monitored the serum metabolome of a large sample of animals over time. This approach enables the investigation of time-dependencies and the identification of early metabolic alterations associated with chronic epilepsy, characterized by the persistence of spontaneous recurrent seizures (SRSs) and the establishment of long-lasting network structural and functional alterations (Curia et al., 2008; Di Liberto et al., 2018; Rho and Boison, 2022). Furthermore, we exploited a phenobarbital (PB) treatment paradigm - widely used in preclinical protocols for drug response stratification - to explore the serum metabolomic profile associated with drug resistance in experimental TLE, an area that remains poorly investigated. Using ^1^H NMR-based serum metabolomics, the study aims to uncover temporal patterns with potential translational relevance for biomarker discovery.

## 2 Materials and methods

### 2.1 Animals

Two-month-old male Wistar rats (body weight 200–250 g) were obtained from Envigo S.r.l. (Indianapolis, IN, USA). In this study we included only male animals to reduce biological variability associated with hormonal fluctuations, which can influence seizure susceptibility and behavioural response, thereby ensuring greater experimental homogeneity. The animals were housed in pairs under controlled environmental conditions, including a 12 h light/dark cycle (lights on from 08:00 to 20:00), ambient temperature maintained between 22 °C and 24 °C, and relative humidity of 50% ± 10%. Food and water were available *ad libitum*. During a 7-day acclimatization period, the rats were regularly handled to reduce stress. All experimental procedures and animal care practices were conducted in accordance with ARRIVE guidelines 2.0 and the European Directive 2010/63/EU. The study protocol was approved by the Animal Welfare Committee of the University of Palermo and authorized by the Italian Ministry of Health (Rome, Italy; authorization no. 761/2021-PR).

### 2.2 Pilocarpine-induced model of TLE

The pilocarpine-induced model of TLE was employed to longitudinally investigate epileptogenesis and epilepsy progression. Following acclimatization, animals were randomly allocated to experimental groups using the online tool Randomizer.org. Ten animals were randomly assigned to the control group and 30 animals to pilocarpine-treated group. Status epilepticus (SE) was induced using a lithium–pilocarpine protocol (Di Liberto et al., 2018). Briefly, rats were pretreated intraperitoneal (i.p.) with lithium chloride (127 mg/kg; Sigma-Aldrich, 310468) 14–16 h before receiving scopolamine methyl bromide (1 mg/kg; Sigma-Aldrich, S8502), a peripheral muscarinic antagonist that does not cross the blood–brain barrier (BBB), administered to reduce the peripheral side effects of pilocarpine. Thirty minutes later, SE was triggered by pilocarpine i.p. administration (10 mg/kg; Sigma-Aldrich, P6503). If SE did not develop within 30 min, additional doses of pilocarpine (10 mg/kg each) were administered at 30-min intervals until SE onset, up to a maximum cumulative dose of 100 mg/kg. SE onset was defined as the occurrence of at least two seizures of stage 4 or 5 according to the Racine scale (Racine, 1972) within a 30-min period, as previously reported (Ferlazzo et al., 2016). Three pilocarpine-treated animals were excluded because they did not develop SE. SE was terminated 90 min after onset, by i.p. administration of a pharmacological cocktail consisting of diazepam (10 mg/kg; Mylan Generics, 5 mg/mL, Viatris), phenobarbital (25 mg/kg; Luminale, 200 mg/mL, Substipharm), and scopolamine hydrobromide (1 mg/kg; Sigma-Aldrich, S1875). The same treatment was repeated 3 h later. To reduce mortality and limit SE-related complications, supportive care was provided. Animals received 1.5 mL of lactated Ringer’s solution subcutaneously (s.c.), followed 2 h later by an injection of saline. During the subsequent 5 days, rats were rehydrated daily with 1.5 mL of 5% dextrose solution (s.c.) in the morning and 2 mL of saline (i.p.) 6 h later to promote recovery and counteract weight loss. Additionally, palatable food was provided to encourage feeding (Guarino et al., 2022). Five animals died during SE or within 72 h post-SE. Of the remaining animals, two were excluded during the chronic phase due to the absence of SRSs, in accordance with established protocols (Di Liberto et al., 2018; Costa et al., 2021).

Lastly, one control animal died between four-eight weeks post-SE.

### 2.3 Video monitoring and drug resistance classification

SRSs were detected via continuous video monitoring starting 8 weeks after pilocarpine induction and lasting for six consecutive weeks, using a wired ZOSI surveillance system equipped with eight Full HD (1080p, 2.0 MP) cameras and an 8-channel DVR with H.265+ compression to optimize data storage and transmission. Ten weeks post-induction, PB treatment was initiated and continued for 2 weeks with twice-daily i.p. administrations. An initial loading dose of 25 mg/kg was followed by maintenance doses of 15 mg/kg. Classification into drug-sensitive and drug-resistant groups was based on previously reported criteria (Bankstahl et al., 2012). Rats were classified as drug-sensitive if PB treatment resulted in complete suppression of seizures or in a reduction of at least 50% in seizure frequency during the post-treatment phase compared with the pre-treatment baseline. Animals not meeting these criteria were categorized as drug-resistant. PB was selected as exclusive anti-seizure medication for drug resistance assessment since it represents the most widely validated and employed compound in experimental epilepsy protocols for responsiveness stratification, allowing reproducibility across studies (Leite and Cavalheiro, 1995; Bankstahl et al., 2012).

Based on these criteria, animals were classified after 12 weeks post SE into two subgroups: 11 drug-sensitive and nine drug-resistant animals. Specifically, seizure frequency was calculated as the total number of SRSs divided by the number of days of video monitoring during pre-treatment, treatment and post-treatment phase (2 weeks each). In the drug-sensitive group, the mean SRS frequency decreased from 0.25 seizures/day before the treatment to 0.12 seizures/day during treatment, followed by a mean frequency of 0.16 seizures/day in the post-treatment phase. In contrast, the drug-resistant group showed no reduction with a mean SRS frequency of 0.16 seizures/day before treatment, which increased to 0.21 seizures/day during PB administration and reached 0.43 seizures/day after treatment.

To minimize potential bias, video monitoring scoring and seizure frequency quantification were performed by experimenters blinded to the experimental group allocations and treatment protocols.

### 2.4 Blood sampling and serum preparation

**FIGURE 1:**
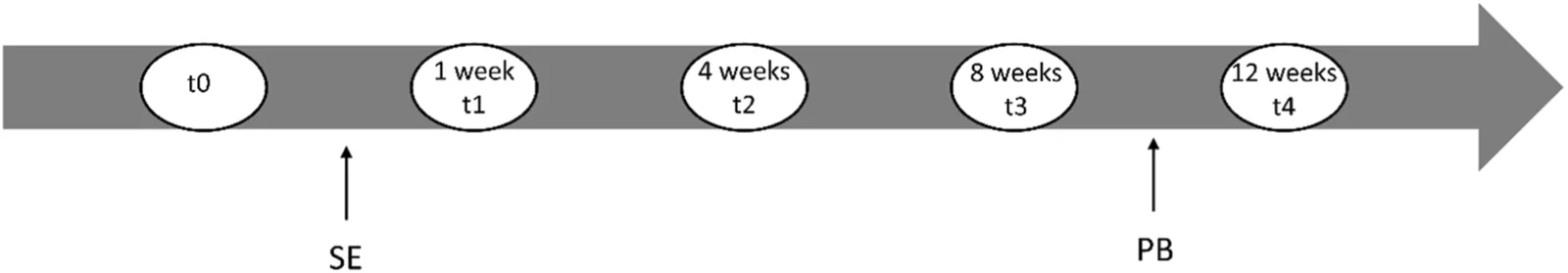
Schematic representation of the workflow of the present study. SE and PB interventions refer only to the epilepsy group, whereas the sampling times are the same for the control and epilepsy groups.

Approximately 1 mL of blood was collected from the lateral tail vein of each rat and transferred into EDTA-free tubes. Samples were left at room temperature for 30 min to allow complete clotting, then centrifuged at 3,000 *g* for 15 min. The resulting serum was carefully separated, collected, and stored for subsequent analyses. Blood sampling was performed longitudinally at five predefined timepoints (Figure 1) to cover the different stages of epileptogenesis and disease progression (Curia et al., 2008; Di Liberto et al., 2018; Lévesque et al., 2021); t0 represented the baseline, before SE induction. t1, 1 week post-SE, corresponded to the acute phase, characterized by prolonged seizure activity and incomplete recovery of consciousness. t2, 4 weeks post-SE, corresponded to a phase of progressive structural and network reorganization in the brain. t3, 8 weeks post-SE, reflected the chronic phase, with the appearance of SRSs originating in the temporal lobe. Finally, t4, 12 weeks post-SE, was dedicated to the assessment of drug (PB) responsiveness, following the protocols described above.

Only animals from which high-quality blood samples were successfully collected at all five timepoints were included in the metabolomic analyses. According to this criterion, only one animal, belonging to the drug-resistant group, was excluded because the blood samples collected at t2 and t3 were affected by haemolysis, preventing reliable assessment. Then, the total number of animals used for the metabolomics analysis was 28: control animals (n = 9) and pilocarpine-treated animals (n = 19), including drug-sensitive (n = 11) and drug-resistant (n = 8) subgroups.

### 2.5 NMR sample preparation

NMR samples were prepared according to standard procedures (Ghini et al., 2023). Frozen samples were thawed at room temperature and shaken before use. A total of 90 µL of each serum sample was added to 90 µL of a sodium phosphate buffer (70 mM Na_2_HPO_4_; 20% (v/v) ^2^H_2_O; 0.025% (v/v) NaN_3_; 0.8% (w/v) sodium trimethylsilyl [2,2,3,3–^2^H_4_]propionate (TSP) pH 7.4).The mixtures were homogenized by vortexing for 30 s and a total of 160 μL of each mixture was transferred into a 3.00 mm NMR tube (Bruker BioSpin) for analysis.

### 2.6 NMR spectra acquisition and processing

^1^H-NMR spectra for all samples were acquired using a Bruker 600 MHz spectrometer (Bruker BioSpin) operating at 600.13 MHz proton Larmor frequency and equipped with a 5 mm PATXI ^1^H-^13^C-^15^N and ^2^H-decoupling probe including a z-axis gradient coil, an automatic tuning-matching (ATM) and an automatic and refrigerated sample changer (SampleJet, Bruker BioSpin). A BTO 2000 thermocouple served for temperature stabilization at the level of approximately 0.1 K at the sample. Before measurement, samples were kept for 5 min inside the NMR probe head, for temperature equilibration at 310 K.

Serum is a heterogeneous mixture composed of metabolites as well as macromolecules like proteins and lipoproteins. Due to its intrinsic characteristic, for each serum sample, three monodimensional ^1^H NMR spectra were acquired with water peak suppression and different pulse sequences that allowed the selective observation of different molecular components:

- a standard NOESY (Nuclear Overhauser Effect Spectroscopy) 1Dpresat (noesygppr1d.comp; Bruker BioSpin) pulse sequence, using 64 scans, 98,304 data points, a spectral width of 18,028Hz, an acquisition time of 2.7s, a relaxation delay of 4 s and a mixing time of 0.1 s. This pulse sequence is designed to obtain a spectrum in which both signals of metabolites and high molecular weight molecules (lipids and lipoproteins) are visible;
- a standard CPMG (cpmgpr1d.comp; Bruker BioSpin) pulse sequence, using 64 scans, 73,728 data points, a spectral width of 12,019 Hz and a relaxation delay of 4 s. This pulse sequence is designed for the selective observation of small molecule components in solutions containing macromolecules;
- a standard diffusion-edited (ledbgppr2s1d.comp; Bruker BioSpin) pulse sequence, using 64 scans, 98,304 data points, a spectral width of 18,028 Hz and a relaxation delay of 4 s. This pulse sequence is designed for the selective observation of macromolecule components in solutions containing small molecules; the resulting spectrum is generally made up only of the lipid, lipoprotein and protein signals.

Free induction decays were multiplied by an exponential function equivalent to a 0.3 Hz line-broadening factor before applying Fourier transform. Transformed spectra were automatically corrected for phase and baseline distortions and calibrated. All the serum spectra were calibrated to the signal of glucose doublet at δ 5.24 ppm, using TopSpin 3.5 (Bruker BioSpin).

### 2.7 Signal assignment and quantification

The metabolites, whose peaks in the spectra were well defined and resolved, were assigned using the Chenomx Software. The assignment of the lipid resonances and lipoprotein-related parameters was performed using literature data; not assigned resonances were labelled as lipid 1–7.

To date, there is no validated approach available for automatically quantifying metabolites and lipoproteins in animals (Di Paco et al., 2026). Therefore, the relative quantification of the NMR signals was performed using CPMG and diffusion-edited spectra. The CPMG spectra, which allow the selective observation of low-molecular-weight molecules, were used for metabolite signal integration. In contrast, diffusion-edited spectra, which selectively detect high-molecular-weight components, were used to integrate lipoprotein resonances. The relative concentrations of the various parameters were calculated by integrating the corresponding signals in defined spectral range, using a home-made tool for signal integration. The integration ranges were manually defined based on the spectral assignment and kept constant across all samples. The resulting peak areas were normalized to the total spectral area of each spectrum, excluding the residual water resonance. The integral of each peak in a given sample is only compared with the corresponding peak in the other samples using the same integral range. This approach ensures that any potential bias related to different relaxation times on the NMR signal intensity does not affect the validity of the relative quantitative comparisons between groups.

### 2.8 Statistical analysis

The panel of the relative quantification of metabolites and lipid/lipoprotein resonances was used to perform multivariate and univariate statistical analyses. Multivariate analyses were conducted using the MetaboAnalyst6.0 platform (Pang et al., 2024). Orthogonal partial least squares discriminant analysis (OPLS-DA) was applied to cluster the two groups based on their overall metabolic fingerprints and to identify potential differences between them at each time point. Q^2^ and *R* ^2^ values represent the predictability and goodness of fit, respectively. The statistical significance for each OPLS-DA model was assessed via permutation test (n = 100).

Univariate statistics were performed using the “R” software. For pairwise comparison within control and epilepsy groups, the paired Wilcoxon signed-rank test was used to analyse the differences between the samples at each time point with respect to t0. For comparison between animals of different group the Mann-Whitney U test was used. p-values ≤ 0.01 were considered statistically significant. All obtained p-values were adjusted for multiple tests using False Discovery Rate Correction (FDR) according to the Benjamini-Hochberg method. The effect size, using the Cliff’s delta (Cd) formulation, was also calculated to aid in the identification of the meaningful signals giving an estimation of the magnitude of the separation between the different groups. The magnitude is assessed using the thresholds provided in Romano and Coll. (Romano et al., 2006), i.e., |δ| < 0.147 “negligible (1)”, |δ| < 0.33 “small (2)”, |δ| < 0.474 “medium (3)”, otherwise “large (4)”.

To test whether temporal profiles differ between experimental groups, we exploited linear mixed-effects models (LMM), using the “lmer” function in “lme4” library. Prior to statistical modeling, metabolite relative concentrations were normalized to baseline by dividing the value at each timepoint by its corresponding baseline (t0) value for each animal, expressing data as relative fold-changes over time. Each metabolite was modeled with Phenotype (control vs. epilepsy), Time (treated as a continuous variable), and their two-way interaction (Phenotype x Time) as fixed effects, while subject identity was included as a random intercept to account for repeated measures. Analysis of Variance (ANOVA, “anova” function, “lmerTest” library) was used to derive p-values for the interaction term. To assess at which specific timepoints significant differences occurred between groups, follow-up *post hoc* pairwise comparisons were conducted using estimated marginal means via the “emmeans” library, with Tukey’s adjustment for multiple comparisons.

All *post hoc* power calculations were performed using G*Power software at a two-tailed significance, level of alpha = 0.05. The sample sizes provided ≥ 80% power to detect medium-to-large intra-group effects (d_z_ ≥ 0.70) and large inter-group differences (d_z_ ≥ 1.15), Supplementary Table S1.

## 3 Results

The combined use of the different NMR sequences described in Section 2.6 enabled the identification and relative quantification of 30 metabolites and 18 lipoprotein-derived groups (Table 1).

**TABLE 1:** Statistical significance of metabolite changes quantified by ^1^H-NMR spectroscopy across experimental groups.

Metabolite/lipid fraction	Control group								Epilepsy group							
	t0 vs. t1		t0 vs. t2		t0 vs. t3		t0 vs. t4		t0 vs. t1		t0 vs. t2		t0 vs. t3		t0 vs. t4	
	p-value	r	p-value	r	p-value	r	p-value	r	p-value	r	p-value	r	p-value	r	p-value	r
Isoleucine	0.73	1	1.00	1	0.82↓	2	0.25↓	2	0.21↓	2	0.31↑	2	0.42	1	0.029↑	4
Leucine	0.82	1	0.82	2	0.50↓	3	0.07↓	4	0.86	1	0.98	1	0.98	1	0.00005↑*	4
Valine	0.57	1	0.65	2	0.91	2	0.16↓	3	0.24↓	3	0.54	1	0.35	1	0.045	3
Lactate	1.00	1	0.25	3	0.012↓	4	0.20	3	0.95↓	1	0.95↓	1	0.33	1	0.54↓	2
Alanine	0.02↓	4	0.008↓	4	0.008↓	4	0.008↓	4	0.087	2	0.24	2	0.80	1	0.61	1
Acetate	0.57	1	0.65	1	0.91	1	0.50	1	0.49	1	0.12	1	0.12	2	0.40	1
Glutamine	1.00	1	0.16	2	0.50	1	0.16↓	2	0.0001↑*	4	0.0002↑*	4	0.00001↑*	4	0.00005↑*	4
Pyruvate	0.098↓	3	0.02↓	4	0.008↓	4	0.074↓	4	0.54	1	0.71	1	0.17	2	0.74	2
3-Hydroxybutyrate	0.008↑	3	0.0391↑	3	0.16↑	1	0.43	1	0.00003↑*	4	0.00001↑*	4	0.002↑*	4	0.00005↑*	4
Lysine	0.57	1	0.43	1	0.074	2	0.02↓	4	0.073↑	3	0.02↑	3	0.40↑	2	0.11↑	2
acetoacetate	0.0273↑	2	0.055↑	2	0.13↑	2	0.57	1	0.00005↑*	4	0.00001↑*	4	0.009↑*	3	0.00001↑*	4
Glutamate	0.43	1	0.07	3	0.0273↓	4	0.098↓	4	0.90	1	0.27	2	0.11	3	0.08	3
Methionine	0.20	3	0.57	1	0.16↓	2	0.05↓	4	0.98	1	0.92	1	0.37	1	0.10↑	3
Creatine	0.04	2	0.01↓	4	0.039↓	3	0.0039↓	4	0.00042↓*	4	0.04↓	2	0.016↓*	3	0.026↓*	3
Glucose	0.05↓	3	0.30	1	0.65	2	0.43	1	0.18	2	0.31	2	0.17	2	0.007↓*	4
Ornithine	1.00	1	0.16	2	0.039	3	0.05↓	4	0.12↑	2	0.169↑	2	0.26	2	0.087↑	3
Choline	0.01↑	3	0.07↑	4	0.57	2	0.57	1	0.60	1	0.23↑	2	0.05↑	2	0.04↑	3
Glycine	0.43	1	0.05	2	0.07	2	0.008↓	4	0.001↑*	4	0.83	1	0.95	1	0.00034↑*	4
Proline	0.25	2	0.65	2	0.91	1	0.36	3	0.036	2	0.74↑	1	0.89	1	0.74	1
Methanol	0.65	2	0.25	2	0.02	3	0.13	3	0.44	2	0.95	1	0.023	3	0.018*	3
Threonine	0.91	2	0.57	2	0.73	2	0.3	3	0.096	2	0.29	2	0.001↓*	4	0.073	3
Tyrosine	0.91	1	0.36↓	3	0.16↓	3	0.098↓	4	0.073	2	0.51	1	0.087	2	0.008↓*	4
Phenylalanine	0.65	2	0.098	3	0.02↓	4	0.05↓	4	0.37	1	0.68	1	0.26	1	0.26	1
Histidine	0.57	3	0.25	3	0.07	4	0.07	4	0.57	1	0.77	1	0.50	1	0.89	1
Formate	0.73	2	0.30	2	0.25	1	0.13	1	0.087	1	0.62	1	0.08	3	0.20	1
Mannose	1.00	1	0.36	2	0.91	1	0.36	2	0.004↑*	2	0.54	2	0.029↑*	3	0.036↑*	3
Citrate	0.73	2	0.30	2	0.25	2	0.039↓	4	0.04↑	3	0.42	2	0.92	1	0.86	1
Acetone	0.008↑	3	0.04↑	3	0.098↑	4	0.30↑	2	0.00001↑*	4	0.00002↑*	4	0.0001↑*	4	0.00001↑*	4
Dimethylsulfone	1.00	2	0.65	1	0.25	2	0.16↓	3	0.045	2	0.036↓	3	0.0003↓	4	0.011↓	4
O-Phosphocholine	0.73	2	0.16↓	3	0.0039↓	4	0.04↓	4	0.001↓*	4	0.008*	4	0.008↓*	4	0.001↓*	4
Cholesterol (CH_3_)	0.43	1	0.65	1	0.05↓	2	0.16↓	2	0.005↑*	3	0.95	1	0.012↑*	2	0.001↑*	3
Lipoprotein (CH_3_)	0.82	1	0.91	1	0.82	1	0.3	1	0.26	1	0.00002↓*	4	0.06	2	0.18	1
Lipoprotein (CH_2_)	0.57	2	0.098	1	0.098↑	4	0.04↑	3	0.002↓*	4	0.04↓	3	0.0001↓*	4	0.003↓*	4
–CO–CH_2_–CH_2_–(βCH_2_)	0,2	2	0.25	2	0.074↑	3	0.04↑	3	0.002↓*	4	0.40	1	0.001↓*	4	0.001↓*	4
Lipid 1	0.91	2	0.82	1	0.3	3	0.07	3	0.002↑*	4	0.001↑*	4	0.00004↑*	4	0.007↑*	4
Cholesterol (CH_2_)	0.57	2	0.16	2	0.02↓	4	0.0078↓	4	0.011↑*	3	0.74	1	0.98	1	0.41	2
-CH_2_-CH = CH-	1.00	1	0.91	1	0.65	2	0.0039	4	0.001↓*	4	0.001↓*	4	0.00001↓*	4	0.001↓*	4
NAG	0.05	4	0.65	2	1.00	1	0.73	1	0.89	1	0.032	2	0.032*	2	0.00001↑*	4
Lipid 2	0,06	3	0.098	2	0.027↓	4	0.012↓	4	0.002↑*	4	0.37	1	0.073↑	3	0.00005↑*	4
–CO–CH_2_–CH_2_–(αCH2)	0.36	1	0.30	2	0.16↑	3	0.04↑	3	0.003↓*	4	0.40	1	0.001↓*	4	0.00034↓*	4
= CH-CH_2_-CH =	0.57	1	0.73	1	0.25	2	0.02↑	4	0.073↓	3	0.004↓*	3	0.029↓*	3	0.012↓*	2
N(CH_3_)_3_-phospholipids	0.65	1	1.00	1	0.25	2	0.73	1	0.54	1	0.00004↓*	4	0.40	1	0.33	2
Lipid 3	0.36	2	0.36	2	0.13↓	3	0.03↓	4	0.003↑*	4	0.018↑*	3	0.001↑*	4	0.001↑*	4
Lipid 4	0.82	1	0.82	2	0.91	2	0.30	1	0.12↑	2	0.00003↑*	4	0.023↑*	3	0.80	1
Lipid 5	0.74	1	0.43	2	0.25	3	0.03↓	4	0.0001↑*	4	0.0003↑*	4	0.002↑*	4	0.007↑*	3
Lipid 6	1.00	1	0.91	1	0.43	2	0.13	3	0.005↑*	4	0.004↑*	3	0.00005↑*	4	0.001↑*	4
Lipid 7	0.73	1	0.50	1	0.25↑	3	0.07↑	4	0.003↓*	4	0.31↓	1	0.00021↓*	4	0.001↓*	4
-CH = CH-	0.91	2	0.73	1	0.20↑	3	0.03↑	4	0.003↓*	4	0.00027↓*	4	0.00027↓*	4	0.004↓*	4

The table reports the longitudinal variations for Control and Epilepsy groups. Statistical comparisons were performed between the baseline (t0) and each subsequent time point (t1, t2, t3, t4). Uncorrected p-values: statistical significance was determined using paired Wilcoxon signed-rank test. Values highlighted in green indicate high statistical significance (p ≤ 0.01). * p-value <0.05 after FDR correction. Cliff’s Delta magnitude ranking values (r) are also reported: negligible (1), small (2), medium (3), large (4); values highlighted in the table indicate a large effect size (threshold ≥3). ↑/↓ indicates an increase/decrease in the median values of relative concentrations in t1, t2, t3 or t4 samples with respect to t0.

**FIGURE 2:**
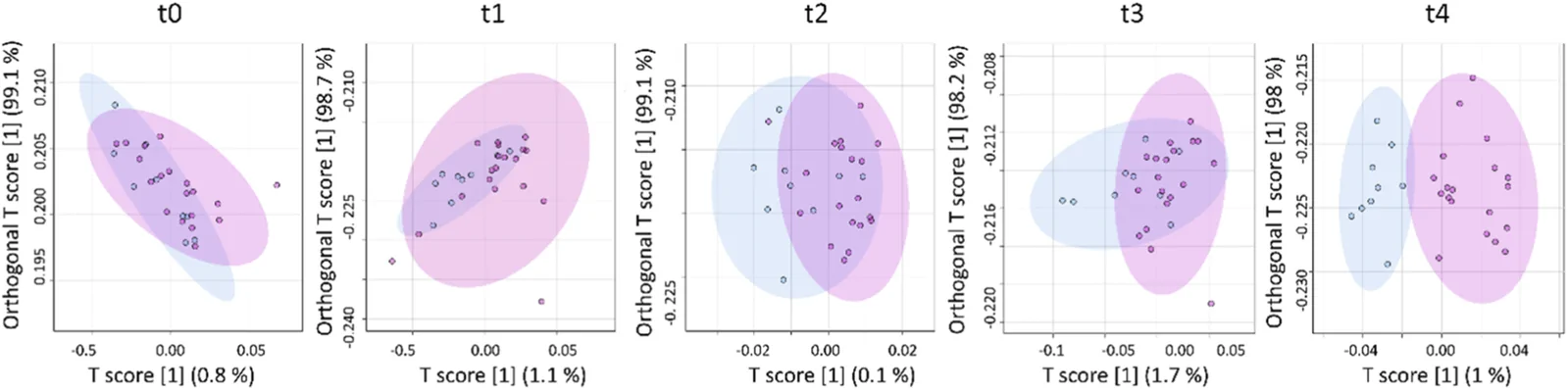
OPLS-DA score plots. Blue dots, control group; magenta dots, epilepsy group and superimposed 95% confidence ellipses.

**FIGURE 3:**
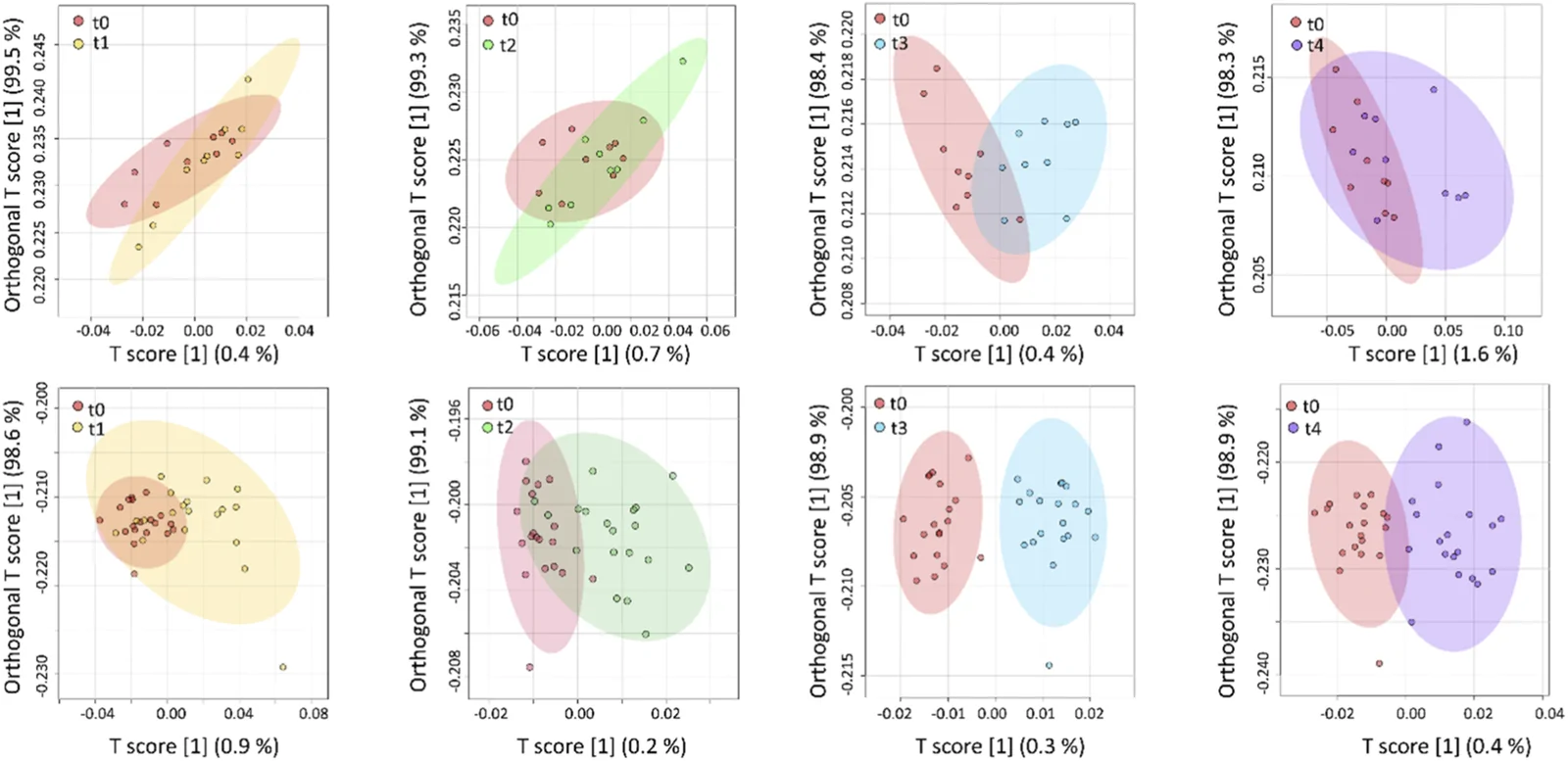
OPLS-DA score plots. Upper panel, control group; lower panel, epilepsy group. Colour coding: red dots (t0); yellow dots (t1); green dots (t2); cyan dots (t3); purple dots (t4). The same colour coding is used for the 95% confidence ellipses.

The serum samples from 28 animals, collected at the five time points (t0 – t4) were analysed via statistical analysis: nine rats in the control group and 19 rats on the epilepsy group, for a total of 140 samples. To visualize the global metabolic shifts, we performed OPLS-DA. When comparing the epilepsy and control groups at discrete time points, a progressive divergence was observed (Figure 2; Supplementary Table S2). While the metabolic space occupied by the two groups overlapped at the early stages (t1), a significant separation emerged starting at 4 weeks post-SE (t2) and became more pronounced at t3 and t4. The longitudinal evolution within each group (Figure 3; Supplementary Table S3) further confirmed this trend. Although a certain degree of discrimination emerges at t3 (but not at t4) for control group, in the epilepsy cohort the serum metabolome underwent a continuous remodeling from t2 to t4.

**FIGURE 4:**
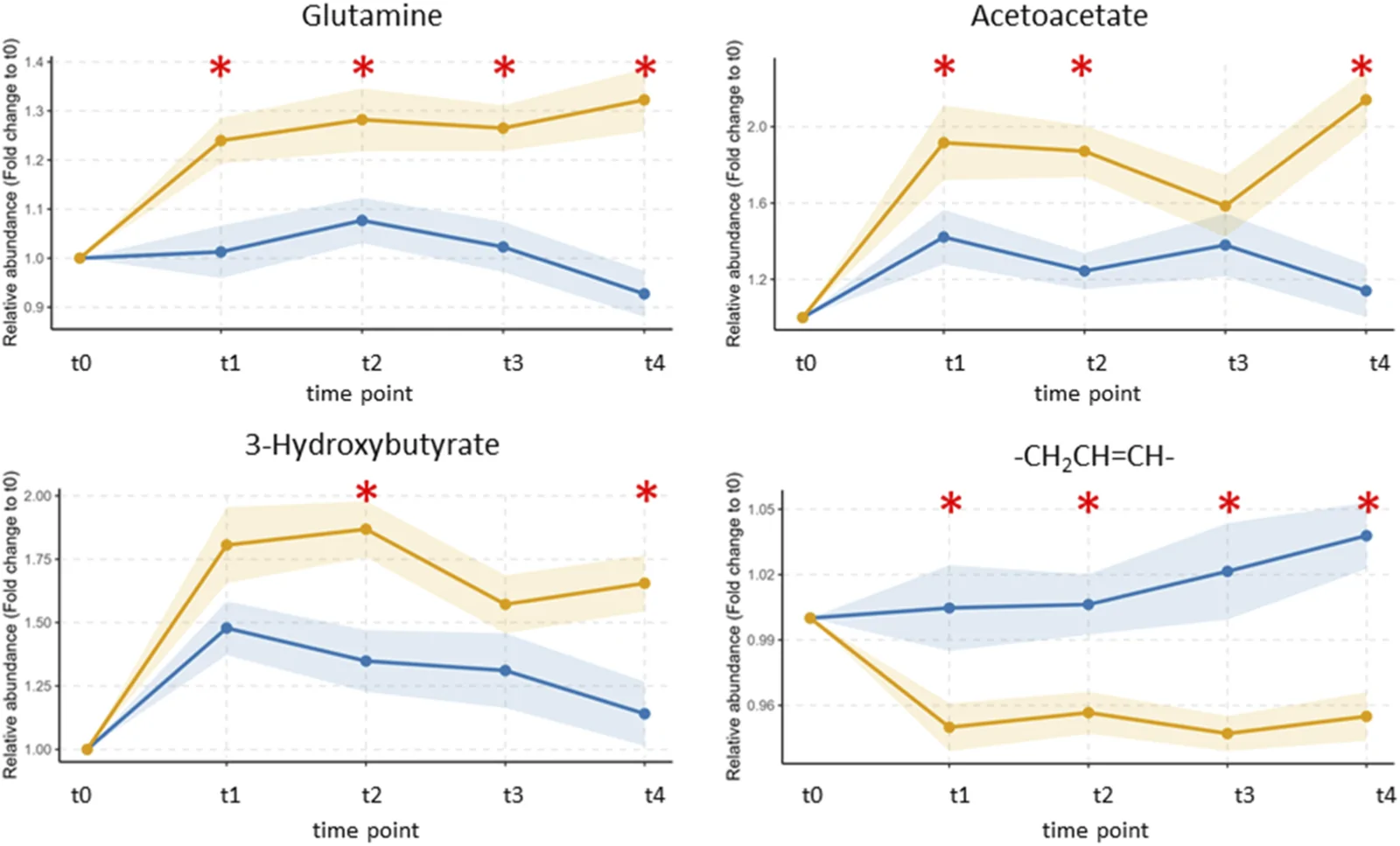
LMM trajectory plots of the relative abundance (fold change to t0) for glutamine, acetoacetate, 3-hydroxybutyrate and the lipid group -CH_2_CH = CH-. Blue trace: control; orange trace: epilepsy. * indicates p-value <0.05 for *post hoc* pairwise comparisons between control and epilepsy groups.

Univariate analysis (Wilcoxon signed-rank test and Cliff’s delta) was employed to pinpoint specific molecular changes relative to the baseline (t0). In the epilepsy group, several metabolites exhibited sustained dysregulation across the chronic phase (Table 1). An increase in 3-hydroxybutyrate, acetoacetate, and Gln levels, concomitant with a decrease in O-phosphocholine, was observed from t1 through t4; the boxplots of their concentration trends over time are reported in Supplementary Figure S1. Additionally, most lipid components underwent extensive remodeling over time (box plot reported in Supplementary Figure S2). Consistently, Variable Importance in Projection (VIP) scores (Supplementary Tables S4, S5) calculated for OPLS-DA for t3 and t4 time points of Figures 2, 3 (bottom panel) identified as key drivers of the discrimination multiple lipid/lipoprotein fractions. Longitudinal analysis using LMM confirmed a significantly different temporal trajectory between the two groups for 3-hydroxybutyrate, acetoacetate, Gln, and almost all the lipid fractions (Figure 4; Supplementary Table S6). In contrast, no statistically significant difference in temporal trend was observed for O-phosphocholine between control and epileptic animals.

To extrapolate the specific effect of the PB treatment, we selected in the epilepsy group the set of metabolites/lipoprotein components that were significantly affected in the comparison t0 vs. t4 but not for t0 vs. t3, and that do not show the same behavior in the control group (uncorrected p-value ≤ 0.01 and Cliff’s delta ≥3). These were leucine, glucose, glycine, NAG and lipid 2 (Table 1, last column). The boxplots of their concentration trends over time are reported in Supplementary Figure S3.

**FIGURE 5:**
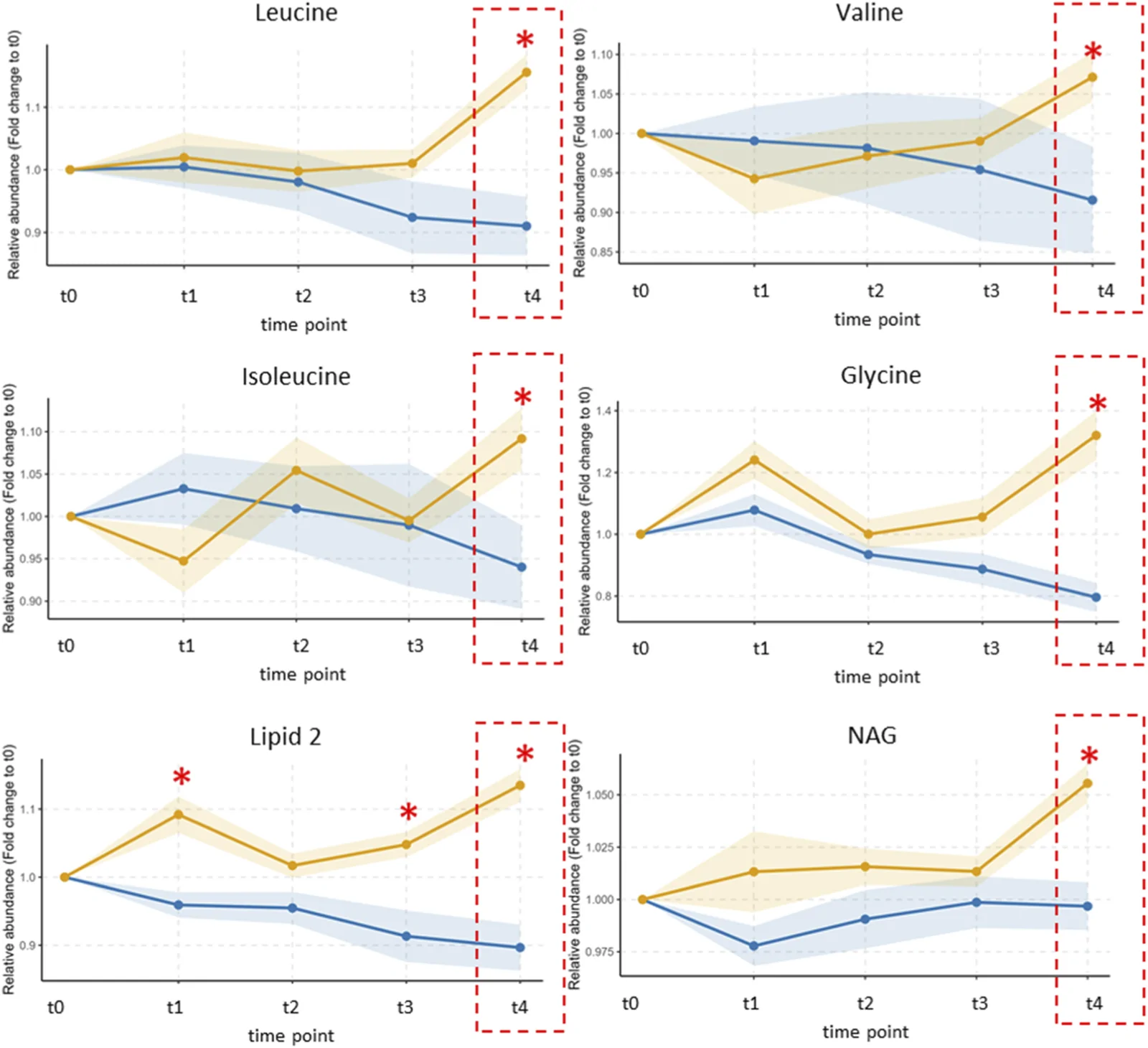
Effect of the PB treatment: LMM trajectory plots of the relative abundance (fold change to t0) for leucine, valine, isoleucine, glycine, lipid 2 and NAG. Blue trace: control; orange trace: epilepsy. * indicates p-value <0.05 for *post hoc* pairwise comparisons between control and epilepsy groups. The red boxes highlight the behaviour at t4.

Furthermore, the LMM analysis confirmed that the temporal pattern observed for leucine was also shared by the other branched-chain amino acids (BCAAs), isoleucine and valine, while consistently validating the trajectories of glycine, NAG, and lipid 2 (Figure 5; Supplementary Table S6).

**FIGURE 6:**
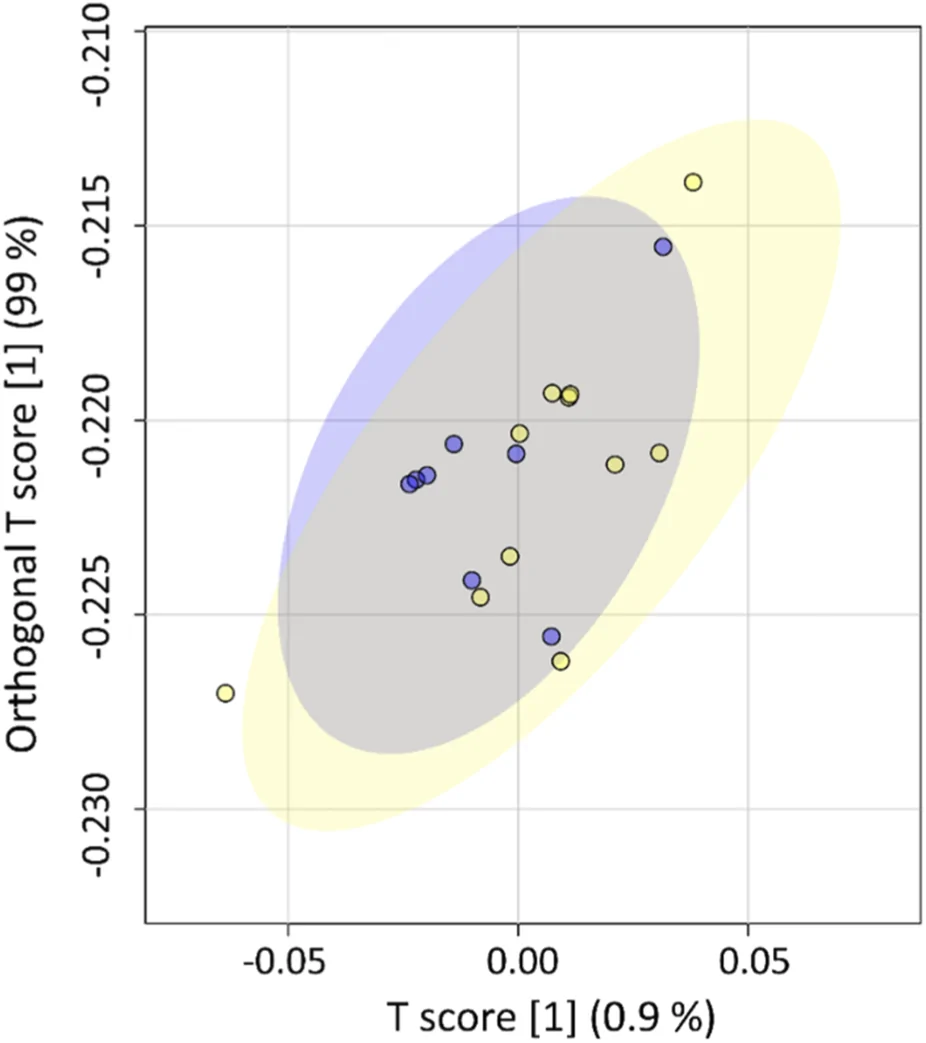
OPLS-DA score plot of t4 samples from epilepsy group. Purple dots, resistant rats; yellow dots, sensitive rats and superimposed 95% confidence ellipsoids.

Following the inclusion criteria described in Section 2.3, the epileptic rats were divided in drug-sensitive (n = 11) and drug-resistant (n = 8) animals. The longitudinal metabolite analysis of Table 1 was repeated considering each group separately (Supplementary Table S7). A comparison with Table 1 revealed that both cohorts followed the same trends previously described for the entire population; however, some localized loss of statistical significance was observed, likely attributable to the reduced sample size in the stratified groups. In order to gain deeper insights into the divergence between the cohorts, inter-group differences were evaluated at every sampling point from t0 through t4 (Supplementary Table S8). No significant differences capable of predicting a divergent metabolic profile were observed at any time point prior to PB administration. Regarding the samples collected at t4, the NMR fingerprints of the two groups do not significantly differ from each other; in fact, the samples cluster within the same metabolic space in the multivariate analysis (Figure 6; Supplementary Table S9). Creatine is the sole feature that distinguishes resistant from sensitive animals with statistically significant decrease in drug-resistant animals (Supplementary Table S8) with an unadjusted p-value = 0.001 and an area under the ROC curve of 0.9 (Supplementary Figure S4). The fact that a single feature can be statistically significant in univariate testing without driving separation in the global multivariate projection is not uncommon (Saccenti et al., 2014).

## 4 Discussion

^1^H NMR profiling revealed a systemic metabolic response related to chronic epilepsy. The longitudinal analysis revealed a progressive metabolic shift characterized by a significant increase in ketone bodies, specifically 3-hydroxybutyrate and acetoacetate. This observation suggests a systemic alteration in energy substrate utilization, likely shifting toward fatty acid oxidation. Furthermore, the sustained decrease in O-phosphocholines points toward a remodeling of cell membrane phospholipids, a phenomenon often associated with neuroinflammatory processes and altered lipid signaling in the epileptic brain. However, a decreasing trend is observed for O-phosphocholine also in the control group, although significance is enriched only at t3. The elevated concentration of glutamine has been reported also by others and associated to dysfunctional neurotransmission (Wang et al., 2021).

The observed decrease in dimethylsulfone (DMSO_2_) (with significance only at t3 and t4), alongside the increase in glycine (with significance only at t0 and t4), provides further evidence of a systemic metabolic signature of epilepsy. Although these alterations reached statistical significance only at specific time points, they are consistent with previous findings (Antmen et al., 2025).

Metabolomic literature in TLE is highly heterogeneous, with significant divergence between Mass Spectrometry (MS) and NMR platforms, as well as inconsistent biomarker signatures across human and animal cohorts (Meier et al., 2024; Antmen et al., 2025; Avalos et al., 2025). To our knowledge, only one study has characterized the plasma metabolic trajectory in a rat model of lithium-pilocarpine-induced TLE (Antmen et al., 2025). In that study, samples had been collected at 48 h, 1 week, and 6 weeks post- SE. Notably, that research employed a cross-sectional design, analyzing distinct cohorts (n = 3–8) at each time point rather than a longitudinal follow-up of the same subjects. For comparison purposes with our results, the temporal dysregulation of significant metabolites identified by Antmen et al. is summarized in Supplementary Figure S5.

Although our experimental design was structured to characterize drug resistance, at t4, despite the clear divergence in clinical outcomes (sensitive vs. resistant), both groups occupied nearly identical metabolic spaces in multivariate OPLS-DA models (Figure 6). This “metabolic convergence” suggests that the systemic burden of chronic epilepsy and the pharmacological effect of PB are the dominant drivers of the serum metabolome, effectively masking the more subtle molecular signatures of drug resistance. This aligns with recent studies using more sensitive platforms, such as MS, which have struggled to identify robust peripheral biomarkers for pharmacoresistance in similar models (Henke et al., 2020; Engelke et al., 2021; Wen et al., 2021). In general, while the application of metabolomics to elucidate the mechanisms of drug resistance represents a burgeoning subfield (Lim et al., 2025), existing animal model data (Henke et al., 2020; Engelke et al., 2021; Lum et al., 2023), are exclusively limited to MS-based platforms.

The only exception in our findings to the systemic similarity between drug-sensitive and -resistant animals was creatine, which exhibited a significant difference between the two phenotypes only after group stratification (p = 0.001). Creatine plays a pivotal role in the ATP/phosphocreatine buffering system, and its divergence at the final time point may reflect differences in energy metabolism between responders and non-responders. The fact that this difference was masked when analyzing the epilepsy group as a whole, underscores the necessity of stratified longitudinal designs to uncover biomarkers that are otherwise obscured by inter-individual variability between drug-sensitive and drug-resistant phenotypes and positions creatine as a compelling feature of interest for future targeted investigations. Finally, while ^1^H NMR provides a robust and reproducible overview of high-abundance metabolites, its inherent sensitivity limits the detection of low-concentration molecules (Takis et al., 2019; Vignoli et al., 2019) (e.g., specific lipids or neurotransmitters) that might further distinguish the two phenotypes.

Crucially, as the present study is based on serum measurements, the observed alterations primarily reflect whole-body metabolic processes, including major contributions from peripheral tissues such as the liver, skeletal muscle, and adipose tissue. Therefore, while these systemic alterations may influence energy substrate availability to the central nervous system, they should not be interpreted as direct readouts of cerebral metabolism. Future studies integrating brain tissue metabolomics or localized imaging approaches with multi-omic peripheral profiling will be essential to bridge this gap and uncouple central resistance mechanisms from systemic pathological effects.

## 5 Conclusion

In conclusion, our untargeted ^1^H NMR approach demonstrates the robust capability of serum metabolomics to characterize the systemic signatures of epilepsy. By employing a longitudinal design, we confirmed several key metabolic shifts that align with and expand upon previous findings (Antmen et al., 2025). A central finding of this study is the remarkable similarity between the metabolic fingerprints of pharmacoresistant and pharmacosensitive animals at the systemic level. Therefore, our data suggest that while the serum metabolome is an excellent reporter of the pathological state (epilepsy vs. control), it may have inherent limitations as a predictor of pharmacological resistance, pointing toward the need for integrating peripheral data with localized brain-tissue metabolomics or more sensitive multi-omic approaches.

## Data Availability

The raw data supporting the conclusions of this article will be made available by the authors, without undue reservation.
