# Supplementary material for: Longitudinal 1H NMR metabolomics of serum in a chronic temporal lobe epilepsy model reveals systemic metabolic remodeling
Source: Front Mol Biosci. 2026 Aug 31;13:1882162. doi: 10.3389/fmolb.2026.1882162 (PMC13572219; doi:10.3389/fmolb.2026.1882162)
Supplement: Supplementary file 1 [file DataSheet1.pdf]

Longitudinal ^{1}H NMR Metabolomics of Serum in a Chronic Temporal Lobe Epilepsy Model Reveals Systemic Metabolic Remodeling

Veronica Ghini[1], Lucrezia Cosottini[1], Miriana Scordino[2], Nicolò Ricciardi[2], Giulia Urone[2], Giuditta Gambino[2], Valentina Di Liberto[2*], Paola Turano[1,3*]

[1]Department of Chemistry, University of Florence, Sesto Fiorentino, Italy

[2]Department of Biomedicine, Neuroscience and Advanced Diagnostics (BIND), University of Palermo, Palermo, Italy

[3]CERM, University of Florence, Sesto Fiorentino, Italy

*** Correspondence:**
Corresponding Authors
paola.turano@unifi.it
valentina.diliberto@unipa.it

Supplementary Material

Supplementary Table 1. Post Hoc Power Analysis Summary Table.

Comparison Type	Statistical Test	Sample Size (N)	Effect Size Threshold	Achieved Power (1−β)	Minimum detectable effect size
Intra-Control Group *(longitudinal)*	Wilcoxon signed-rank *(paired data)*	N = 9	d_z = 1.20	83.4%	Large effects
Intra-Epilepsy Group *(longitudinal)*	Wilcoxon signed-rank *(paired data)*	N = 19	d_z = 0.70	81.1%	Medium-to-large effects.
Inter-Group<br>*(control vs epilepsy)*	Mann-Whitney U *(independent samples)*	N = 9<br>vs.<br>N = 19	d_z = 1.15	80.2%	Large effects
Inter-Group<br>*(drug-sensitive vs -resistant)*	Mann-Whitney U *(independent samples)*	N = 11<br>vs.<br>N = 8	d_z = 1.25	80.7%	Large effects

Supplementary Table 2. Statistical validation and quality parameters for supervised (Orthogonal PLS-DA) multivariate models reported in Figure 6 (main text); cum=cumulative.

**Timepoint**	**Model**	**R^2X(cum)**	**R^2Y(cum)**	**Q^2(cum)**	**Permutation p(R^2Y)**	**Permutation p(Q^2)**
t0	control vs. epilepsy	0.991	0.071	0.032	0.23	0.17
t1	control vs. epilepsy	0.987	0.166	0.077	0.039	0.043
t2	control vs. epilepsy	0.997	0.357	0.166	0.035	0.02
t3	control vs. epilepsy	0.982	0.292	0.337	<0.01	<0.01
t4	control vs. epilepsy	0.989	0.752	0.650	<0.01	<0.01

Supplementary Table 3. Statistical validation and quality parameters for supervised (Orthogonal PLS-DA) multivariate models reported in Figure 7 (main text); cum=cumulative.

**Group**	**Model**	**R^2X(cum)**	**R^2Y(cum)**	**Q^2(cum)**	**Permutation $p(R^2Y)$**	**Permutation $p(Q^2)$**
Control	t0 vs. t1	0.995	0.072	0.008	0.44	0.08
Control	t0 vs. t2	0.993	0.046	0.0004	0.57	0.11
Control	t0 vs. t3	0.996	0.676	0.411	0.02	<0.01
Control	t0 vs. t4	0.983	0.261	0.289	0.03	0.01
Epilepsy	t0 vs. t1	0.991	0.374	0.217	< 0.01	< 0.01
Epilepsy	t0 vs. t2	0.998	0.610	0.497	< 0.01	< 0.01
Epilepsy	t0 vs. t3	0.997	0.889	0.708	< 0.01	< 0.01
Epilepsy	t0 vs. t4	0.996	0.776	0.667	< 0.01	< 0.01

Supplementary Table 4. Top 15 VIP scores for the OPLS-DA models (t3 and t4) of Figure 6 (main text)

t3			t4		
	V1	V2		V1	V2
Lipoprotein(CH2)	6.69640239	4.96883213	Lipoprotein(CH2)	6.45564683	4.94955842
Lipid 3	2.24557218	2.80045507	Lipid 3	2.698477	2.80226422
-CH=CH-	1.096933	1.15380725	-CH=CH-	1.1911242	1.1818919
Lipid 2	0.56823631	0.85491772	Lipid 2	0.85212516	0.88983412
–CO–CH2–CH2–(aCH2)	0.54911787	0.49610561	–CO–CH2–CH2–(aCH2)	0.53881176	0.4813716
–CO–CH2–CH2–(bCH2)	0.50902074	0.54715605	–CO–CH2–CH2–(bCH2)	0.50029553	0.53177437
Lactate	0.46754825	0.30198682	NAG	0.41044119	1.31446487
Lipid 1	0.40376271	0.56821827	=CH-CH2-CH=	0.40987324	0.88648294
Lipid 6	0.34657308	0.50174066	Lipid 1	0.37587454	0.55060348
=CH-CH2-CH=	0.27949946	0.88073968	Lipid 6	0.36531961	0.49833368
3-Hydroxybutyrate	0.25318862	0.15701576	Glutamine	0.32610387	0.19836953
Lipid 5	0.22408115	0.73389063	Glucose	0.29105586	0.31939471
-CH2-CH=CH-	0.20612244	0.60506664	Acetatoacetate	0.25537934	0.11065612
N(CH3)3_phospholipids	0.18602686	0.55717246	3-Hydroxybutyrate	0.25536711	0.15778475
Glucose	0.17925588	0.37086488	Lipid 5	0.24213314	0.72010883

Supplementary Table 5. Top 15 VIP scores for the OPLS-DA models (t0 vs. t3 and t0 vs. t4) of Figure 7 (main text)- epilepsy group.

t0 vs. t3			t0 vs. t4		
	V1	V2		V1	V2
lipoprotein(CH2)	6.688902	4.967713	lipoprotein(CH2)	6.411409	4.925073
Lipid 3	1.909552	2.817188	Lipid 3	2.516587	2.848657
-CH=CH-	1.327014	1.160004	-CH=CH-	1.091436	1.164165
Lactate	0.611809	0.324037	Lipid 2	1.005977	0.892872
3-hydroxybutyrate	0.540691	0.14227	–CO–CH2–CH2–(aCH2)	0.614699	0.482779
–CO–CH2–CH2–(aCH2)	0.490989	0.493716	3_hydroxybutyrate	0.601939	0.141571
Lipid 1	0.490848	0.563009	NAG	0.59221	1.312498
lipoprotein(CH3)	0.454415	3.32965	Glucose	0.551838	0.334397
Glutamine	0.441806	0.191627	Acetatoacetate	0.551284	0.095024
–CO–CH2–CH2–(bCH2)	0.430512	0.541759	–CO–CH2–CH2–(bCH2)	0.541356	0.532187
Lipid 2	0.406259	0.859801	Lipid 6	0.417344	0.500204
Lipid 6	0.403942	0.497192	Glutamine	0.41298	0.19531
=CH-CH2-CH=	0.394576	0.882084	=CH-CH2-CH=	0.374238	0.881075
-CH2-CH=CH-	0.343223	0.609015	Lipid 1	0.354868	0.556962
Acetatoacetate	0.297524	0.080846	lipoprotein(CH3)	0.253295	3.3517

Supplementary Table 6. Results from the LMM analysis. First column: p-values for the comparison between the control and epilepsy groups evaluating the "Phenotype x Time" interaction. Second-fourth columns: p-values for post hoc pairwise comparisons at each time point.

Metabolite	p-value Interaction (Phenotype X Time)	t1	t2	t3	t4
Isoleucine	0.007	0.134	0.426	0.924	0.009
Leucine	0.00004	0.768	0.731	0.091	0.000005
Valine	0.015	0.457	0.874	0.576	0.02
Lactate	0.062	0.815	0.407	0.001	0.305
Alanine	0.005	0.002	0.054	0.0001	0.002
Acetate	0.661	0.829	0.193	0.122	0.916
Glutamine	0.00005	0.006	0.012	0.003	0.000005
Pyruvate	0.026	0.174	0.073	0.001	0.056
3-Hydroxybutyrate	0.094	0.069	0.004	0.146	0.005
Lysine	0.0005	0.092	0.109	0.020	0.00002
Acetatoacetate	0.020	0.034	0.007	0.376	0.00003
Glutamate	0.00002	0.530	0.059	0.0008	0.00005
Methionine	0.0002	0.639	0.866	0.039	0.0007
Creatine	0.016	0.066	0.446	0.320	0.049
Glucose	0.069	0.004	0.741	0.285	0.148
Ornithine	0.002	0.250	0.089	0.019	0.0004
Choline	0.006	0.114	0.219	0.664	0.017
Glycine	0.00006	0.068	0.452	0.057	0.00000006
Proline	0.482	0.305	0.232	0.590	0.335
Methanol	0.898	0.639	0.052	0.857	0.956
Threonine	0.019	0.117	0.711	0.002	0.037
Tyrosine	0.194	0.543	0.758	0.374	0.414
Phenylalanine	0.011	0.798	0.225	0.036	0.045
Histidine	0.034	0.100	0.436	0.103	0.016
Formate	0.475	0.602	0.093	0.816	0.249
Citrate	0.071	0.216	0.023	0.298	0.031
Acetone	0.349	0.382	0.021	0.206	0.304
Dimethylsulfone	0.983	0.465	0.209	0.308	0.904
O-Phosphocholine	0.458	0.211	0.635	0.230	0.830
Cholesterol(CH_3)	0.0006	0.010	0.625	0.001	0.0001
Lipoprotein(CH_3)	0.656	0.328	0.003	0.260	0.556
Lipoprotein(CH_2)	0.000004	0.003	0.052	0.000008	0.0000008
$–CO–CH_2–CH_2–(\beta CH_2)$	0.000003	0.002	0.129	0.000003	0.0000001
Lipid 1	0.00008	0.004	0.018	0.00006	0.000009

Cholesterol(CH_2)	0.0006	0.016	0.417	0.0008	0.00004
-CH_2-CH=CH-	0.00002	0.002	0.005	0.00004	0.000007
NAG	0.023	0.046	0.155	0.404	0.001
Lipid 2	0.000003	0.0002	0.070	0.0001	0.0000000004
–CO–CH2–CH2–(αCH_2)	0.000003	0.005	0.204	0.000006	0.0000001
=CH-CH_2-CH=	0.002	0.095	0.097	0.013	0.0003
$N(CH_3)_3$-phospholipids	0.254	0.932	0.020	0.085	0.458
Lipid 3	0.00002	0.001	0.031	0.00005	0.00000008
Lipid 4	0.348	0.685	0.175	0.344	0.340
Lipid 5	0.0002	0.002	0.009	0.0004	0.000002
Lipid 6	0.00004	0.033	0.170	0.001	0.00002
Lipid 7	0.000005	0.015	0.270	0.000005	0.0000006
-CH=CH-	0.00002	0.004	0.018	0.00003	0.0000003

Supplementary Table 7. Statistical significance of metabolite changes quantified by ^{1}H-NMR spectroscopy across experimental groups. The table reports the longitudinal variations for Control, Drug-resistant, and Drug-sensitive groups. Statistical comparisons were performed between the baseline (t0) and each subsequent time point (t1, t2, t3, t4). Uncorrected p-values: statistical significance was determined using paired Wilcoxon signed-rank test. Values highlighted in yellow indicate high statistical significance ($p \leq 0.01$). * p-value < 0.05 after FDR correction. Cliff's Delta magnitude ranking values (r) are also reported: negligible (1), small (2), medium (3), large (4); values highlighted in the table indicate a large effect size (threshold ≥ 3).

	Control group								Drug-resistant								Drug-sensitive							
	t0 vs t1		t0 vs t2		t0 vs t3		t0 vs t4		t0 vs t1		t0 vs t2		t0 vs t3		t0 vs t4		t0 vs t1		t0 vs t2		t0 vs t3		t0 vs t4	
	p-value	r	p-value	r	p-value	r	p-value	r	p-value	r	p-value	r	p-value	r	p-value	r	p-value	r	p-value	r	p-value	r	p-value	r
**Isoleucine**	0.73	1	1.00	1	0.82	2	0.25	2	0.55	2	0.11	4	0.84	1	0.15	4	0.21	2	0.83	1	0.37	1	0.15	3
**Leucine**	0.82	1	0.82	2	0.50	3	0.07	4	0.20	2	0.95	2	0.64	1	0.01*	4	0.64	2	0.90	2	0.97	1	0.007*	4
**Valine**	0.57	1	0.65	2	0.91	2	0.16	3	0.55	3	0.95	2	0.55	1	0.38	2	0.37	2	0.52	2	0.41	3	0.08	3
**Lactate**	1.00	1	0.25	3	0.012	4	0.20	3	0.31	2	1.00	1	0.08	4	0.25	3	0.52	1	0.83	2	0.90	2	0.97	1
**Alanine**	0.02	4	0.008	4	0.008	4	0.008	4	0.74	2	0.55	2	0.31	3	0.74	2	0.08	3	0.24	2	0.37	1	0.70	1
**Acetate**	0.57	1	0.65	1	0.91	1	0.50	1	0.15	3	1.00	1	0.15	4	0.95	2	0.58	1	0.05	3	0.52	1	0.24	2
**Glutamine**	1.00	1	0.16	2	0.50	1	0.16	2	0.01*	4	0.20	3	0.01*	4	0.01*	4	0.007	4	0.0009*	4	0.0009*	4	0.007*	4
**Pyruvate**	0.098	3	0.02	4	0.008	4	0.074	4	0.74	1	1.00	1	0.04	4	0.84	2	0.37	2	0.70	1	0.70	1	0.76	1
**3-Hydroxybutyrate**	0.008	3	0.0391	3	0.16	1	0.43	1	0.01*	4	0.01	4	0.04	4	0.02	4	0.007	3	0.001*	4	0.02	3	0.003*	4
**Lysine**	0.57	1	0.43	1	0.074	2	0.02	4	0.05	4	0.15	3	0.84	1	0.46	2	0.41	2	0.08	2	0.41	2	0.17	3
**Acetatoacetate**	0.0273	2	0.055	2	0.13	2	0.57	1	0.01*	4	0.02	4	0.55	2	0.01*	4	0.009	3	0.001*	4	0.009	4	0.001*	4
**Glutamate**	0,43	1	0.07	3	0.0273	4	0.098	4	0.64	2	0.64	2	0.31	3	0.25	3	0.52	1	0.41	3	0.37	3	0.24	3
**Methionine**	0.20	3	0.57	1	0.16	2	0.05	4	0.46	2	0.74	1	0.55	2	0.25	3	0.32	2	0.58	1	0.76	1	0.32	3
**Creatine**	0.04	2	0.01	4	0.039	3	0.0039	4	0.08	4	0.04	4	0.15	4	0.02	4	0.005	4	0.28	2	0.07	3	0.41	2
**Glucose**	0.05	3	0.30	1	0.65	2	0.43	1	0.46	2	0.11	4	0.38	1	0.15	4	0.41	2	0.83	1	0.32	2	0.05	4
**Ornithine**	1.00	1	0.16	2	0.039	3	0.05	4	0.02*	4	0.74	1	0.11	4	0.20	4	1.00	1	0.17	3	0.97	1	0.32	2
**Choline**	0.01	3	0.07	4	0.57	2	0.57	1	0.20	3	0.84	1	0.20	3	0.15	4	0.76	2	0.17	2	0.17	2	0.21	3

**Glycine**	0.43	1	0.05	2	0.07	2	0.008	4	0.04	4	0.46	1	0.95	1	0.04	4	0.02	4	0.70	1	0.83	1	0.01	4
**Proline**	0.25	2	0.65	2	0.91	1	0.36	3	0.02	4	0.46	2	0.11	3	0.38	3	0.32	2	0.90	1	0.28	2	0.41	3
**Methanol**	0.65	2	0.25	2	0.02	3	0.13	3	0.20	2	0.46	3	0.05	4	0.20	4	0.70	2	0.52	2	0.24	3	0.10	3
**Threonine**	0.91	2	0.57	2	0.73	2	0.3	3	0.38	3	0.74	1	0.01*	4	0.31	3	0.24	2	0.41	2	0.05	4	0.15	3
**Tyrosine**	0.91	1	0.36	3	0.16	3	0.098	4	0.15	2	0.74	2	0.15	4	0.11	4	0.28	2	0.70	1	0.58	1	0.05	3
**Phenylalanine**	0.65	2	0.098	3	0.02	4	0.05	4	0.74	1	0.55	2	0.04	4	0.11	4	0.46	2	0.97	1	0.64	2	0.97	1
**Histidine**	0.57	3	0.25	3	0.07	4	0,07	4	0.95	2	0.20	2	0.15	3	0.64	2	0.64	1	0.41	2	0.52	2	0.52	1
**Formate**	0.73	2	0.30	2	0.25	1	0.13	1	0.08	3	0.31	2	0.11	3	0.64	1	0.52	1	0.08	2	0.46	2	0.17	2
**Mannose**	1.00	1	0.36	2	0.91	1	0.36	2	0.01*	4	0.64	1	0.20	3	0.38	2	0.24	2	0.24	2	0.10	3	0.05	3
**Citrate**	0.73	2	0.30	2	0.25	2	0.039	4	0.02	4	0.55	1	0.84	2	0.84	1	0.46	2	0.05	3	0.90	1	0.76	1
**Acetone**	0.008	3	0.04	3	0.098	4	0.30	2	0.01*	3	0.01	3	0.01*	3	0.01*	1	0.003	1	0.005*	1	0.01	1	0.001*	2
**Dimethylsulfone**	1.00	2	0.65	1	0.25	2	0.16	3	0.01*	4	0.02	4	0.01*	4	0.01*	4	0.76	1	0.46	2	0.02	4	0.21	4
**O-Phosphocholine**	0.73	2	0.16	3	0.0039	4	0.04	4	0.25	2	0.20	3	0.20	4	0.01*	4	0.002	4	0.03	4	0.04	4	0.07	4
**Cholesterol(CH_3)**	0.43	1	0.65	1	0.05	2	0.16	2	0.02*	4	0.46	2	0.01*	4	0.04	4	0.12	3	0.52	1	0.41	1	0.02	3
**Lipoprotein(CH_3)**	0.82	1	0.91	1	0.82	1	0.3	1	0.38	1	0.01	4	0.74	1	0.08	2	0.37	1	0.005*	4	0.007*	4	0.76	1
**Lipoprotein(CH_2)**	0.57	2	0.098	1	0.098	4	0.04	3	0.01*	4	0.38	2	0.01*	4	0.04	4	0.10	4	0.05	3	0.01	3	0.04	4
**–CO–CH_2–CH_2–(βCH_2)**	0,2	2	0.25	2	0.074	3	0.04	3	0.01*	4	0.64	1	0.01*	4	0.02	4	0.10	3	0.37	1	0.05	2	0.03	4
**Lipid 1**	0.91	2	0.82	1	0.3	3	0.07	3	0.01*	4	0.11	4	0.02	4	0.11	4	0.12	4	0.01*	4	0.002*	4	0.04	4
**Cholesterol(CH_2)**	0.57	2	0.16	2	0.02	4	0.0078	4	0.04	4	0.74	2	0.55	2	0.84	1	0.15	3	0.90	1	0.64	1	0.64	3
**-CH_2-CH=CH-**	1.00	1	0.91	1	0.65	2	0.0039	4	0.01*	4	0.01	4	0.01*	4	0.02	4	0.03	4	0.04	3	0.002*	4	0.02	4
**NAG**	0.05	4	0.65	2	1.00	1	0.73	1	0.20	3	0.01	4	0.15	3	0.01*	4	0.32	2	0.52	1	0.17	2	0.003*	4
**Lipid 2**	0,06	3	0.098	2	0.027	4	0.012	4	0.02*	4	0.64	2	0.15	3	0.02	4	0.08	3	0.52	1	0.32	2	0.005*	4
**–CO–CH2–CH2–(αCH_2)**	0.36	1	0.30	2	0.16	3	0.04	3	0.01*	4	0.55	1	0.01*	4	0.02	4	0.12	3	0.32	1	0.07	2	0.014	4
**=CH-CH_2-CH=**	0.57	1	0.73	1	0.25	2	0.02	4	0.02*	4	0.08	3	0.25	3	0.25	2	0.58	2	0.014	4	0.10	3	0.04	3
**N(CH_3)$_3$-phospholipids**	0.65	1	1.00	1	0.25	2	0.73	1	0.46	2	0.01	4	0.08	3	0.15	2	0.90	1	0.005*	4	0.58	2	0.90	1
**Lipid 3**	0.36	2	0.36	2	0.13	3	0.03	4	0.01*	4	0.15	3	0.02	4	0.02	4	0.24	2	0.10	3	0.04	3	0.02	4
**Lipid 4**	0.82	1	0.82	2	0.91	2	0.30	1	0.20	1	0.02	4	0.95	1	0.64	1	0.37	2	0.001*	4	0.001*	4	0.90	1

**Lipid 5**	0.74	1	0.43	2	0.25	3	0.03	4	0.02*	4	0.11	4	0.25	2	0.15	2	0.007	4	0.001*	4	0.003*	4	0.02	4
**Lipid 6**	1.00	1	0.91	1	0.43	2	0.13	3	0.01*	4	0.08	4	0.02	4	0.02	4	0.28	3	0.05	3	0.005*	4	0.02	4
**Lipid 7**	0.73	1	0.50	1	0.25	3	0.07	4	0.01*	4	0.64	1	0.01*	4	0.01*	4	0.15	3	0.41	1	0.03	2	0.03	4
**-CH=CH-**	0.91	2	0.73	1	0.20	3	0.03	4	0.02*	4	0.05	4	0.04	4	0.11	3	0.17	4	0.003*	4	0.005*	4	0.02	4

Supplementary Table 8. Statistical comparison of metabolite levels between Drug-sensitive and Drug-resistant groups at each time point. This table reports the differential metabolic profiles between the two experimental groups (Sensitive vs. Resistant) evaluated at different time points (t0, t1, t2, t3) and following treatment (t4). Uncorrected p-values: statistical significance was determined using Mann-Whitney U test. Values highlighted in yellow indicate high statistical significance ($p \leq 0.01$). * p-value < 0.05 after FDR correction. Cliff's Delta magnitude ranking values (r) are also reported: negligible (1), small (2), medium (3), large (4); values highlighted in the table indicate a large effect size (threshold ≥ 3). / indicates an increase/decrease in the median values of relative concentrations in drug-resistant with respect to grug-sensitive.

	Drug-sensitive vs. drug-resistant									
	t0		t1		t2		t3		t4	
	p-value		p-value		p-value		p-value		p-value	
**Isoleucine**	0.091	4	0.238	3	0.129	3	0.206	3	0.152	3
**Leucine**	0.442	2	0.152	3	0.6	2	0.442	2	0.904	1
**Valine**	0.091	4	0.272	2	0.545	2	0.075	4	0.657	1
**Lactate**	0.129	3	0.091	4	0.351	2	0.492	2	0.062	4
**Alanine**	0.152	3	0.033	4	0.272	2	0.968	1	0.206	3
**Acetate**	0.033	4	0.02	4	0.351	2	0.968	1	0.129	3
**Glutamine**	0.6	2	0.968	1	0.31	2	0.051	4	0.272	2
**Pyruvate**	0.062	4	0.109	3	0.442	2	0.492	2	0.351	2
**3-Hydroxybutyrate**	0.31	2	0.129	3	0.395	2	0.31	2	0.272	2
**Lysine**	0.545	2	1	1	0.904	1	0.152	3	0.177	3
**Acetatoacetate**	0.033	4	0.091	4	0.177	3	0.904	1	0.177	3
**Glutamate**	1	1	0.31	2	0.84	1	0.968	1	0.272	2
**Methionine**	1	1	0.206	3	0.442	2	0.778	1	0.778	1
**Creatine**	0.395	2	0.778	1	0.206	3	0.395	2	0.001	4
**Glucose**	0.395	2	0.395	2	0.109	3	0.272	2	0.272	2
**Ornithine**	0.351	2	0.545	2	0.041	4	0.84	1	0.351	2
**Choline**	0.129	3	0.492	2	0.492	2	0.238	3	0.109	3
**Glycine**	0.6	2	0.6	2	0.545	2	0.31	2	0.206	3
**Proline**	0.075	4	0.152	3	0.041	4	0.968	1	0.778	1
**Methanol**	0.657	1	0.545	2	0.31	2	0.717	1	0.84	1
**Threonine**	0.717	1	0.904	1	0.545	2	0.206	3	0.6	2
**Tyrosine**	0.904	1	0.968	1	0.6	2	0.109	3	0.351	2
**Phenylalanine**	0.442	2	0.442	2	0.545	2	0.051	4	0.31	2
**Histidine**	0.545	2	0.778	1	0.062	4	0.016	4	0.152	3
**Formate**	0.109	3	0.026	4	0.545	2	0.904	1	0.206	3
**Mannose**	0.6	2	0.152	3	0.206	3	0.717	1	0.778	1
**Citrate**	0.904	1	0.091	4	0.177	3	0.442	2	1	1
**Acetone**	0.492	2	0.109	3	0.062	4	0.109	3	0.31	2
**Dimethylsulfone**	0.177	3	0.001	4	0.012	4	0.238	3	0.152	3
**O-Phosphocholine**	0.6	2	0.351	2	0.657	1	0.442	2	0.177	3
**Cholesterol(CH_3)**	0.442	2	0.778	1	0.272	2	0.206	3	0.717	1

**Lipoprotein(CH_3)**	0.717	1	0.968	1	0.904	1	0.395	2	0.904	1
**Lipoprotein(CH_2)**	0.351	2	0.129	3	0.351	2	0.778	1	0.075	4
**–CO–CH_2–CH_2–(βCH_2)**	0.31	2	0.442	2	0.31	2	0.31	2	0.109	3
**Lipid 1**	0.351	2	0.206	3	0.206	3	0.904	1	0.129	3
**Cholesterol(CH_2)**	0.177	3	1	1	0.062	4	0.6	2	0.041	4
**-CH_2-CH=CH-**	1	1	0.177	3	0.657	1	0.778	1	0.968	1
**NAG**	0.091	4	0.84	1	0.442	2	0.395	2	0.84	1
**Lipid 2**	0.075	4	0.6	2	0.206	3	0.395	2	0.109	3
**–CO–CH_2–CH_2–(αCH_2)**	0.395	2	0.657	1	0.152	3	0.31	2	0.062	4
**=CH-CH_2-CH=**	0.206	3	1	1	0.075	4	0.272	2	0.152	3
**N(CH_3)$_3$-phospholipids**	0.177	3	1	1	0.129	3	0.351	2	0.904	1
**Lipid 3**	0.206	3	0.206	3	0.351	2	0.904	1	0.238	3
**Lipid 4**	0.657	1	0.904	1	0.968	1	0.206	3	0.84	1
**Lipid 5**	0.778	1	0.657	1	0.238	3	0.442	2	0.442	2
**Lipid 6**	0.109	3	0.272	2	0.492	2	0.778	1	0.129	3
**Lipid 7**	0.152	3	0.657	1	0.177	3	0.492	2	0.051	4
**-CH=CH-**	0.062	4	0.6	2	0.129	3	0.84	1	0.152	3

Supplementary Table 9. Statistical validation and quality parameters for supervised (Orthogonal PLS-DA) multivariate model reported in Figure 9 (main text); cum=cumulative.

**Group**	**Model**	**R^2X(cum)**	**R^2Y(cum)**	**Q^2(cum)**	**Permutation p(R^2Y)**	**Permutation p(Q^2)**
sensitive vs resistant	t4	0.99	0.0575	-0.219	0.52	0.49

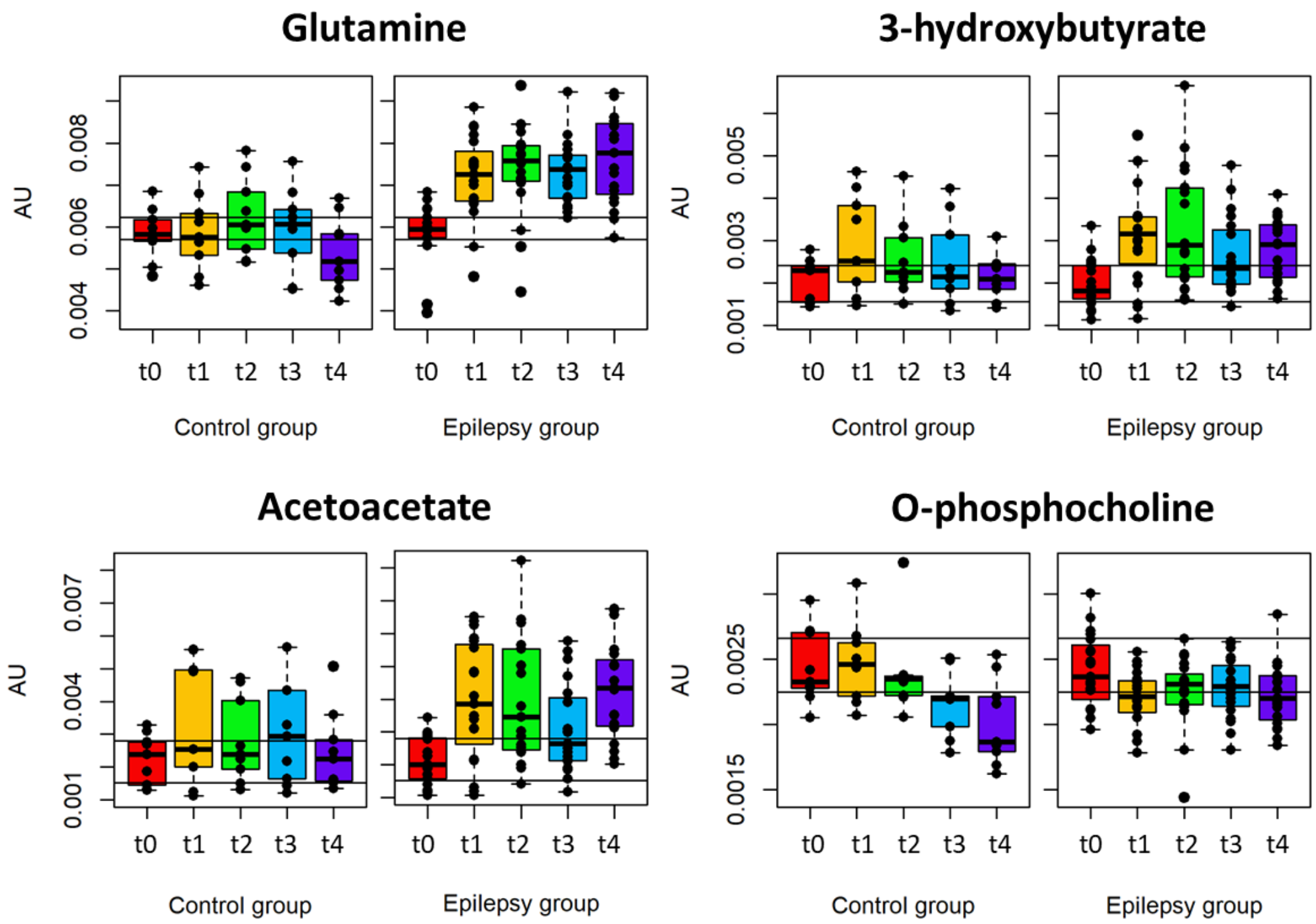

Supplementary Figure 1. Box plots of the concentration trends over time for glutamine, 3-hydroxybutyrate, acetoacetate and O-phosphocholine. In each plot, the two horizontal black lines embrace the relative concentration range at t0, considering all samples (control + epilepsy).

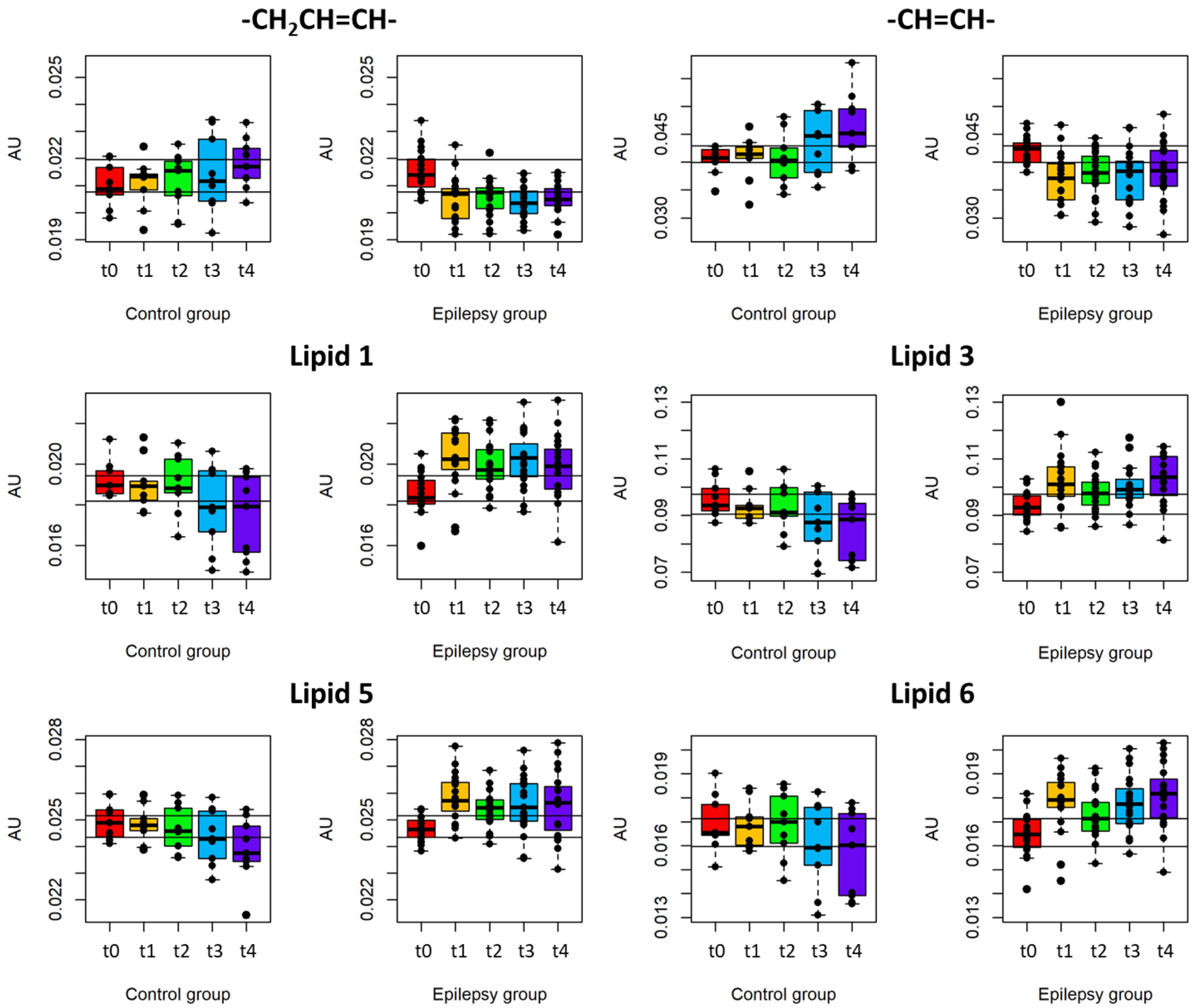

Supplementary Figure 2. Box plots of the concentration trends over time for the lipid components –CH_2–CH=CH–, –CH=CH–, Lipid 1, Lipid 3, Lipid 5, and Lipid 6 in the control and epilepsy groups. In each plot, the two horizontal black lines embrace the relative concentration range at t0, considering all samples (control + epilepsy).

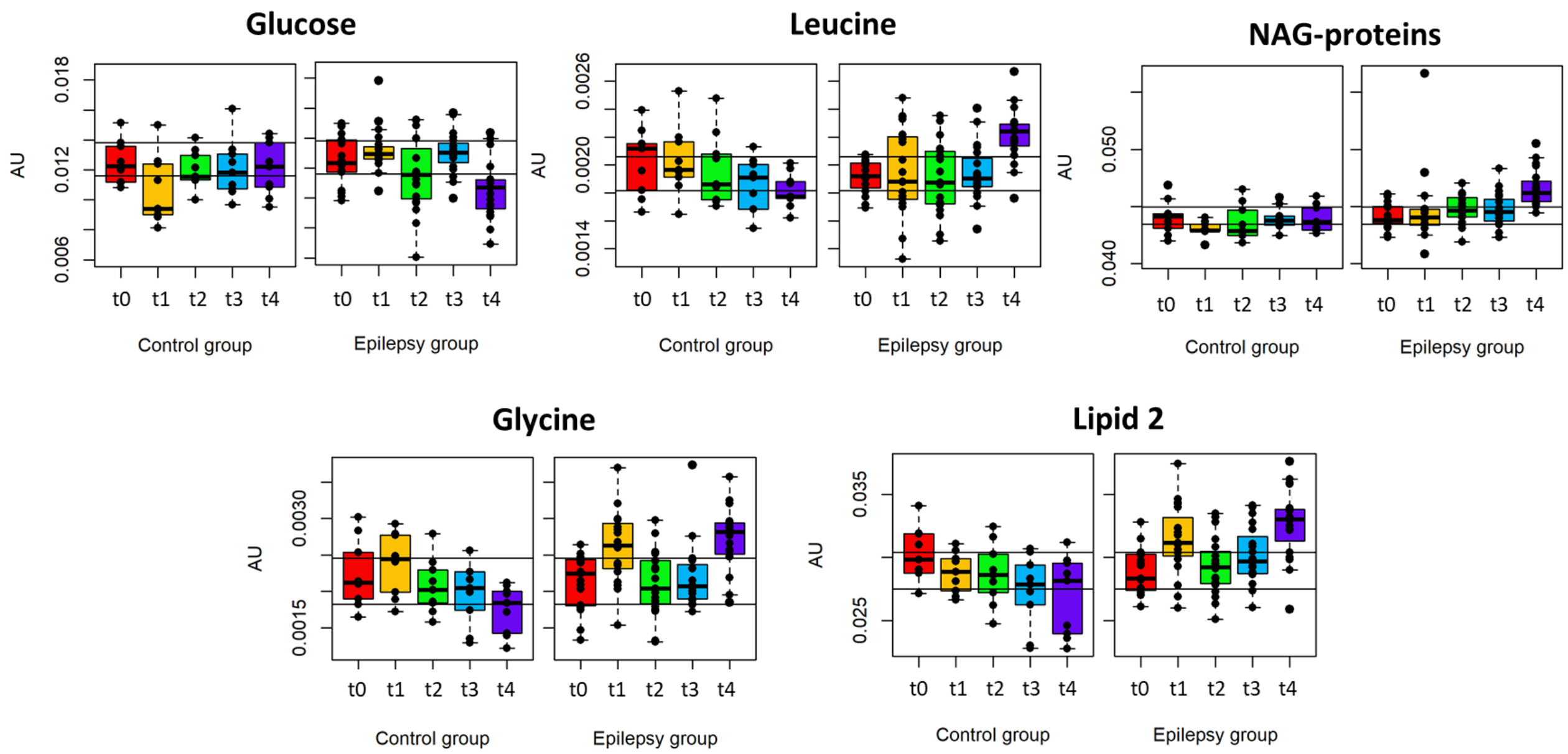

Supplementary Figure 3. Box plots of the concentration trends over time for glucose, leucine, NAG-proteins, glycine and lipid 2. In each plot, the two horizontal black lines embrace the relative concentration range at t0, considering all samples (control + epilepsy).

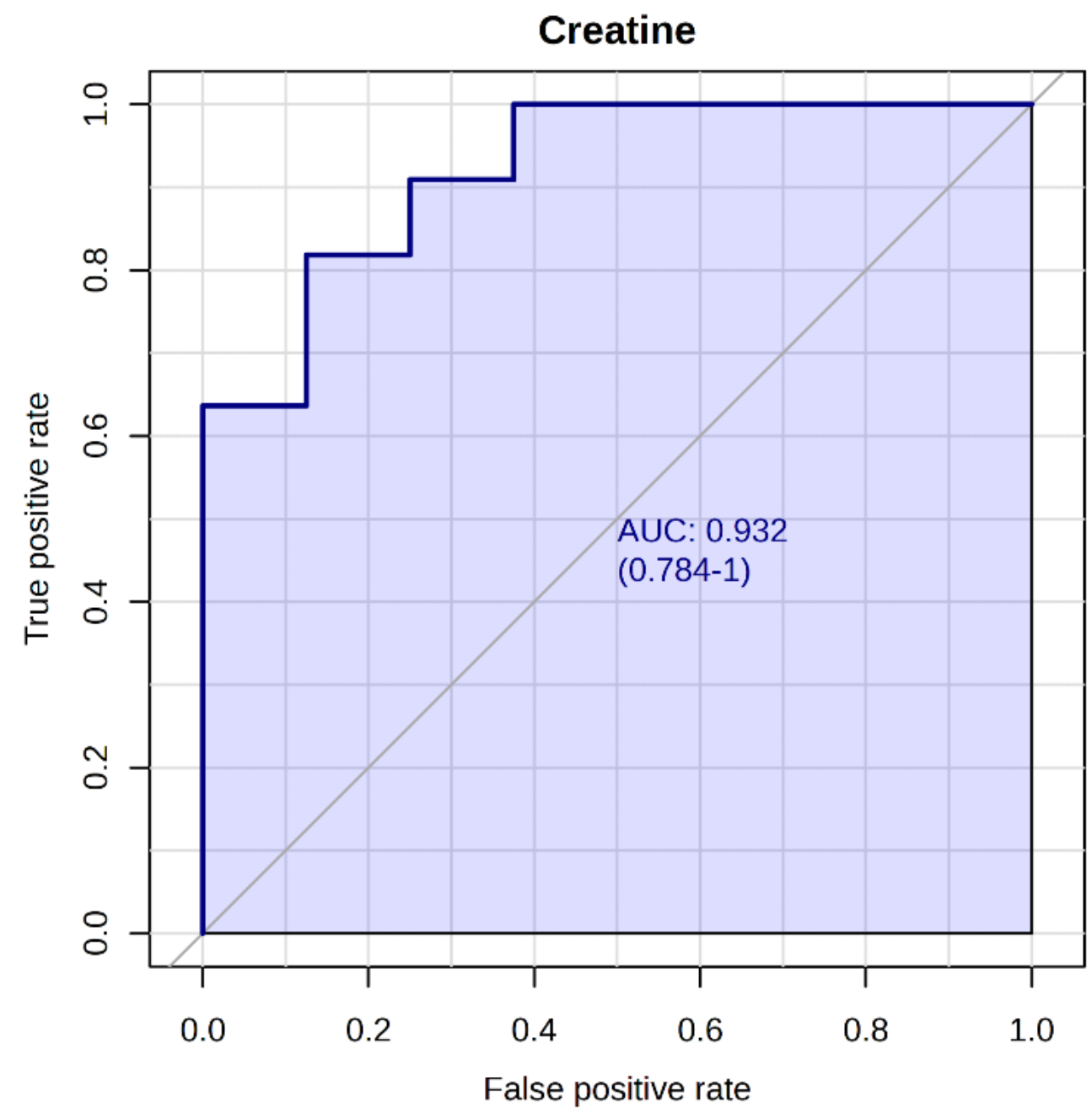

Supplementary Figure 4. ROC curve of creatine for the comparison between resistant and sensitive animals at t4.

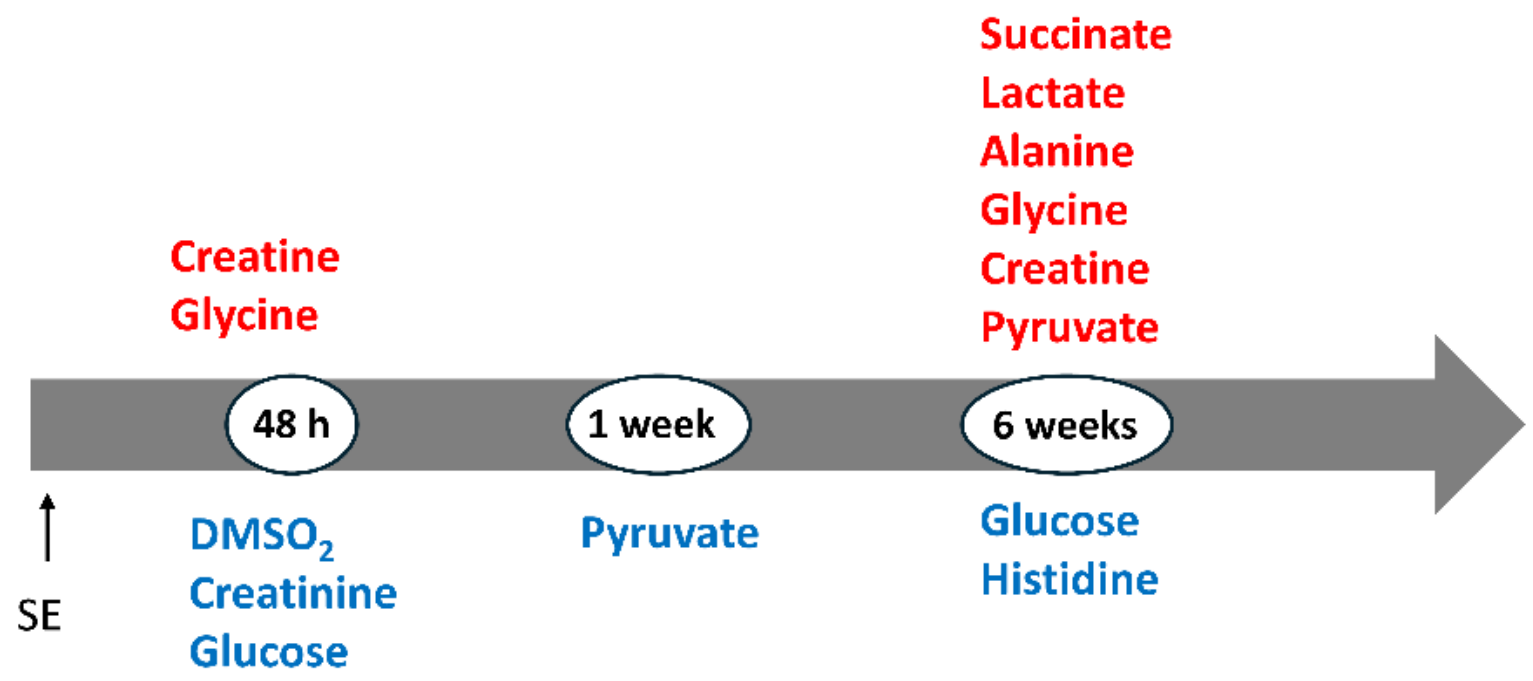

Supplementary Figure 5. Schematic representation of the results reported in (Antmen et al., 2025). Red/blue metabolites are found to have increased/decreased with respect to the initial state, when status epilepticus (SE) has been induced.
